# Cross-sectional survey of off-label and unlicensed prescribing for inpatients at a paediatric teaching hospital in Western Australia

**DOI:** 10.1371/journal.pone.0210237

**Published:** 2019-01-08

**Authors:** Caitlin Landwehr, Jennifer Richardson, Lewis Bint, Richard Parsons, Bruce Sunderland, Petra Czarniak

**Affiliations:** 1 School of Pharmacy and Biomedical Sciences, Curtin University, Perth, Western Australia, Australia; 2 Fiona Stanley Hospital South Metropolitan Health Service, Perth, Western Australia, Australia; 3 School of Occupational Therapy, Social Work and Speech Pathology, Curtin University, Perth, Western Australia, Australia; Universitat Autonoma de Barcelona, SPAIN

## Abstract

**Objectives:**

To evaluate the prevalence of off-label and unlicensed prescribing in inpatients at a major paediatric teaching hospital in Western Australia and to identify which drugs are commonly prescribed off-label or unlicensed, including factors influencing such prescribing.

**Methods:**

A retrospective cross-sectional study was conducted in June, 2013. Patient and prescribing data were collected from 190 inpatient medication chart records which had been randomly selected from all admissions during the second week of February 2013. Drugs were categorised as licensed, off-label or unlicensed, according to their approved Australian registration product information (PI). All drugs were classified according to the Anatomical Therapeutic Chemical (ATC) code.

**Results:**

There were 120 male and 70 female inpatients. The average age was 6.0 years (± 4.7). The study included 1160 prescribed drugs suitable for analysis. The number of drugs prescribed per patient ranged from 1 to 25 with an average of 6.1 (± 4.3). More than half (54%) were prescribed off-label. Oxycodone, clonidine, parecoxib and midazolam were always prescribed off-label. The most common off-label drugs were ondansetron (18.5%), fentanyl (12.9%), oxycodone (8.8%) and paracetamol (6.1%). Many ATC classifications included high off-label proportions especially the genitourinary system and sex hormones, respiratory system drugs, systemic hormonal preparations and alimentary tract and metabolism drugs.

**Conclusions:**

This study highlights that prescribing of paediatric drugs needs to be better supported by existing and new evidence. Incentives should be established to foster the conduct of evidence-based studies in the paediatric population. The current level of off-label prescribing raises issues of unexpected toxicity and adverse drug effects in children that are in some cases severely ill.

## Introduction

Registration of medications in Australia requires assurances of efficacy, quality and safety. The Therapeutic Goods Administration (TGA) is responsible for the licensing and labelling content of medications for use within Australia [1, 2]. However, despite this process medications are often used in either an off-label and occasionally unlicensed manner. Off-label prescribing refers to the use of a drug in a manner different from that for which it is registered [3, 4]. This includes being utilised in the treatment of a different indication, at a different dose or dosage frequency, via a different route of administration and also when it is prescribed for a different age and/or weight to that stated in the registered product information [3, 4]. Unlicensed prescribing includes instances when drugs are prescribed but they have not been licenced for use by the TGA [4, 5]. Modification or reformulation of a licensed drug is also classified as unlicensed prescribing [1, 4, 5].

This practice is highlighted in the prescribing of medications for children. The quantity and range of drugs currently licensed and labelled for use in children is limited and therefore prescribers may resort to off-label or unlicensed use out of perceived clinical necessity. Ethical concerns may limit clinical trials being performed in children leading to a lack of paediatric registration [6]. Such prescribing presents a number of safety concerns. The pharmacokinetics and pharmacodynamics of drugs can be significantly affected by age [6]. In paediatric patients, the two main drug metabolising enzyme systems are the phase I and phase II reactions, which are often different to adults. Phase I reactions, which usually convert the parent drug to a more polar metabolite, involve cytochrome P450 enzymes of which CYP3A4 is the most important and is involved in the metabolism of many drugs including carbamazepine, erythromycin, fentanyl, ketoconazole and nifedipine. The activity of CYP3A4 is very low in neonates, increases in the first 12 months of life and is higher in infants and children than in adolescents and adults. Glucuronidation, a phase II reaction, also varies with age and is reduced in neonates but increases in infants and children. Drugs that undergo predominantly glucuronidation include paracetamol and morphine [7]. The variation in drug metabolism parameters within different age groups highlights the importance of further investigation and consideration for paediatric prescribing [8]. Extrapolation of adult dosing schedules to children may be inappropriate and lead to poor efficacy or adverse effects.

Off-label and unlicensed prescribing is a common occurrence in children’s hospitals globally [9–12]. Past studies conducted in Australia have also found that off-label prescribing is a common occurrence in hospitals. A Tasmanian study involving 300 patients aged ≤ 12 years reported that 31.8% of drugs prescribed in a paediatric ward of a teaching hospital were off-label and that 2 of the 5 reported adverse drug reactions involved drugs used in an off-label manner [2]. It was concluded that the prescribing of medicines off-label was often supported by available evidence and indicated that there was a need for Australian paediatric prescribing guidelines [2].

A study at a major paediatric teaching hospital in Western Australia in 2008 involving inpatients, Emergency Department patients and outpatients ≤ 18 years [1] reported that the clinical setting influenced the level of off-label prescribing. The highest proportions of patients who were prescribed at least one off-label drug were inpatient children aged two to 11 years (85%) and neonates aged zero to 27 days (83%). Overall, the ten most frequently prescribed off-label drugs were ondansetron (13.8%), Painstop Day (10%), salbutamol (7.5%), oxycodone (7.2%), paracetamol (7.1%), midazolam (4.3%), fentanyl (3.1%), Timentin (ticarcillin with clavulanic acid) (2.8%), amoxicillin (2.6%) and flucloxacilin (2.6%).

Since off label or unlicensed prescribing has the potential to be detrimental to patients it is necessary to identify areas of current prescribing practice where this is occurring and to identify its prevalence. In addition, it shows where drug registration data are needed to be expanded by sponsors.

The principal aim of this study was to investigate the prevalence of off-label and unlicensed prescribing for inpatients at Princess Margaret Hospital (PMH). The study also investigated major causative factors behind this practice and aimed to determine if there was any association between off-label and unlicensed prescribing practices and demographic or diagnostic data.

## Methods

A retrospective cross-sectional study was conducted on inpatients at PMH, a 220 bed tertiary teaching paediatric hospital in Western Australia. Data were collected in June, 2013. The study randomly selected (using a web-based program) 215 medication chart records from 595 inpatient cases for the second week of February, 2013. Each record was given a unique re-identifiable code, held by the Chief Pharmacist at PMH. Data collected included date of birth, sex, weight, height (when available), presenting complaint, diagnosis, known allergies, and a full medication profile for each subject. The medication profile included the drug, dose, frequency, indication and route of administration. Drugs were given a separate listing if the same drug was prescribed at a different dose or for a different indication. Also, if the drug was given via more than one route, and the different route resulted in a different classification, the drug was then given two separate listings for ease of analysis. All data were entered into Microsoft Excel.

Of the 215 randomly selected medication charts, six were excluded as the indications in one chart were unclear and for five oncology patients, access to their files was limited at the time of data collection. Other exclusions were medications charts from patients on psychiatric drugs as these were unable to be accessed, incomplete medication charts or medication charts not able to be located at the Patient Information Management Services (PIMS), where the records were held.

Drug exclusions included any gases and inhalations used during surgery such as nitrous oxide and sevoflurane; propofol; blood products; intravenous fluid drips or pushes; oxygen therapy; and any medications given in the Emergency Department.

Following data collection, each prescribed drug was classified as licensed, off-label or unlicensed by consulting the Product Information (PI) from the Therapeutic Goods Administration website [13] and the 2013 eMIMS [14]. The drug was listed as off label if any of the following applied:

1. Dosage: Doses within 10% of the value specified in the PI were considered licensed. This allowed for rounding, and was done to reduce the chance of over estimating the prevalence of off-label use. Doses outside this range (>±10%) were listed as high or low compared to the PI, based on age or weight.
2. Age/ weight: The PI stated that the drug was not approved or recommended for use in children under a specific weight or age.
3. Indication: The PI stated that the drug was indicated for a condition different to the diagnosis.
4. Route: The drug was administered via a route not listed in the PI.

Drugs were classified as unlicensed if they were reformulated, not registered with the TGA, prepared extemporaneously or obtained through the Special Access Scheme (SAS). Under the SAS, if a patient requires access to therapeutic goods that are not listed on the Australian Register of Therapeutic Goods (ARTG), unapproved therapeutic goods may be imported and/ or supplied for a single patient.

Each drug was classified according to the Anatomical Therapeutic Chemical (ATC) code [15].

The age of each subject was classified according to the European Medicines Agency (EMA) classification system which defines neonates as zero to 27 days, infants as 28 days to 23 months, children as two to eleven years, adolescents as 12 to 18 years, and adults as eighteen years and over [16].

Ethics approval was granted by Curtin University Human Research Ethics Committee (Approval number PH-13-13) and Princess Margaret Hospital.

### Statistical analyses

Simple descriptive statistics (frequencies and percentages for categorical variables, means and standard deviations for continuous variables) were used to summarise patient demographic data, and the drugs they were prescribed. Comparisons of the age profile, and the route of administration of the drugs between drugs listed as off-label and unlicensed was performed using Chi-square statistics. Analyses were performed using the SPSS version 20 statistical software. A p-value < 0.05 was taken to indicate a statistically significant association.

## Results

The study evaluated 190 paediatric inpatients of which 120 (63.2%) were male and 70 (36.8%) were female. The average patient age was 6.0 years (± 4.7). Patient weight ranged from 2.0 to 73.0 kg, the average weight was 26.3 kg and the median was 18.8 kg. There were 1160 prescribed drugs suitable for analysis, of which 44.5% (n = 516) were licensed, 54.0% (n = 626) were off-label and 1.6% (n = 18) were unlicensed. The number of drugs prescribed per patient ranged from 1 to 25 with an average of 6.1 (± 4.3).

The most common reasons for off-label prescribing were indication and age (Table 1). With respect to off-label classification regarding dosage, higher doses occurred almost twice as often as lower doses (Table 1). Frequently drugs were classified as off-label for more than one reason. This is demonstrated by a cumulative off-label frequency percentage of 137% of all the drugs classified as off-label (Table 1). The majority of unlicensed medications were extemporaneous preparations (55.6%) or reformulations (38.9%). Prescribing through the SAS was rare (Table 1).

**Table 1:** Major causative factors for drugs to be classified as off-label or unlicensed (n = 626 off-label, n = 18 unlicensed).

Off-label category	Number n (%)	Unlicensed category	Number n (%)
Dosage—High	120 (19.2)	Reformulation	7 (38.9)
Dosage—Low	74 (11.8)	Extemporaneous preparation	10 (55.6)
Indication	371 (59.3)	SAS drug	1 (5.6)
Age	253 (40.4)		
Route of administration	39 (6.2)		

Drugs commonly used off-label included ondansetron, fentanyl, oxycodone and paracetamol (Table 2). Some drugs were always classified as off-label including clonidine, oxycodone, midazolam and parecoxib (Table 2). Drugs commonly unlicensed included dexamethasone and chlorhexidine.

**Table 2:** Frequency and percentage of off-label and unlicensed drug use for the 20 most frequently prescribed drugs (n = 868).

Drug	Prescribed Frequency	Off-label n (%)	Unlicensed n (%)
**Paracetamol**	171	38 (22.2)	0.0
**Ondansetron**	134	116 (86.6)	0.0
**Fentanyl**	94	81 (86.2)	0.0
**Ibuprofen**	90	3 (3.3)	0.0
**Emla** (lignocaine/ prilocaine)	58	1 (1.7)	0.0
**Oxycodone**	55	55 (100.0)	0.0
**Cephazolin**	38	14 (36.8)	0.0
**Morphine**	34	14 (41.2)	0.0
**Dexamethasone**	31	29 (93.6)	2 (6.5)
**Clonidine**	21	21 (100)	0.0
**Amoxicillin**	19	13 (68.4)	0.0
**Timentin** (ticarcillin/ clavulanic acid)	18	14 (77.8)	0.0
**Atracurium**	15	6 (40.0)	0.0
**Flucloxacillin**	15	10 (66.7)	0.0
**Gentamicin**	15	4 (26.7)	0.0
**Parecoxib**	13	13 (100.0)	0.0
**Salbutamol**	13	11 (84.6)	0.0
**Chlorhexidine**	11	2 (18.2)	8 (72.7)
**Midazolam**	11	11 (100.0)	0.0
**Painstop Day** (paracetamol/ codeine phosphate)	11	9 (81.8)	0.0

The highest frequency of off-label prescribing occurred in neonates, although there were no significant differences in the profile of licensed, off-label and unlicensed drugs between age groups (p = 0.39) (Table 3). The most commonly prescribed off-label drugs for each age group were as follows (figures quoted are number and percentages of the off-label drugs administered within each age group: Table 3);

- Neonates—amoxicillin (n = 3; 37.5%) and nystatin (n = 2; 25.0%)
- Infants—fentanyl (n = 17; 14.7%), ondansetron (n = 14; 12.1%) and paracetamol (n = 10; 8.6%)
- Children—ondansetron (n = 86; 21.2%), fentanyl (n = 50; 12.4%) and oxycodone (n = 40; 9.9%)
- Adolescents—ondansetron (n = 16; 16.5%), fentanyl (n = 13; 13.4%) and oxycodone (n = 10; 10.3%)

**Table 3:** Frequency of off label and unlicensed prescribing within each EMEA age classification group, as well as total number of prescriptions per age group. P value was obtained through a Fisher’s Exact test, due to the small number of unlicensed medicines.

	Off-label n (%)	Unlicensed n (%)	Total number of prescriptions	P value
**Neonate** **(Zero- 27days)**	8 (66.7)	0 (0.0)	12	0.3922
**Infant** **(28 days- 23 months)**	116 (50.7)	3 (1.3)	229	
**Children** **(2–11 years)**	405 (54.5)	15 (2.0)	743	
**Adolescents** **(12–18 years)**	97 (55.1)	0 (0.0)	176	

No unlicensed drugs were prescribed for neonates. The three unlicensed drugs prescribed to infants were chlorhexidine (n = 1), diazoxide (n = 1) and phenylephrine (n = 1). The 15 unlicensed drugs prescribed to children included: chlorhexidine (n = 7; 46.7%) and dexamethasone or omeprazole in equal numbers (n = 2; 13.3%).

Overall, while 53.9% of all drugs were classified as off-label, the Fisher’s Exact test showed that off-label and unlicensed rates were strongly associated with the route of administration (p<0.0001). Notably, the prescribing of topical products was much higher for both the licensed and unlicensed categories as shown in Table 4.

**Table 4:** Frequency of prescribing with respect to category based on route of administration. P value was obtained using a Fisher’s Exact test.

	Licensed n (%)	Off-label n (%)	Unlicensed n (%)	P value
**Oral**	253 (51.8)	228 (46.7)	7 (1.4)	< 0.0001
**Injectable**	187 (36.5)	324 (63.3)	1 (0.2)	
**Topical**	72 (75.8)	13 (13.7)	10 (10.5)	
**Inhaled**	4 (6.3)	60 (93.8)	0 (0.0)	

According to the ATC classification, drugs most commonly prescribed overall were for the nervous system, the alimentary tract and metabolism and also anti-infectives (Table 5). Off-label drugs were most often those used for the alimentary tract and metabolism, respiratory system, blood and blood forming organs and sex hormone preparations (Table 5). Unlicensed drugs were most often those used for the cardiovascular system and sensory organs (Table 5).

**Table 5:** Frequency of off label and unlicensed prescribing for each ATC class, and as a percentage of overall prescribing for each ATC class (n = 1160).

ATC Class (n = 1160; 100.0%)	Frequency of off-label prescribing n (%)	Frequency of unlicensed prescribing n (%)	Licensed n (%)
**Alimentary tract and metabolism (n = 225**; 19.4%)	163 (72.4)	10 (4.4)	52 (19.4)
**Blood and blood forming organs** (n = 4; 0.3%)	3 (75.0)	0 (0.0)	1 (25.0)
**Cardiovascular system** (n = 8; 0.7%)	2 (25.0)	2 (25)	4 (50.0)
**Dermatologicals** (n = 7; 0.6%)	2 (28.6)	1 (14.3)	4 (57.1)
**Genitourinary system and sex hormones** (n = 2; 0.2%)	2 (100.0)	0 (0.0)	0 (0.0)
**Systemic hormonal preparations, excluding sex hormones and insulins** (n = 41; 3.5%)	33 (80.5)	2 (4.9)	6 (14.6)
**Anti-infectives** (n = 195; 16.8%)	112 (57.4)	0 (0.0)	83 (42.6)
**Antineoplastic and immunomodulating agents** (n = 9; 0.8%)	6 (66.7)	0 (0.0)	3 (33.3)
**Musculoskeletal** (n = 30; 2.6%)	11 (36.7)	1 (3.3)	18(60.0)
**Nervous system** (n = 608; 52.4%)	273 (44.9)	0 (0.0)	335 (55.1)
**Antiparasitic products, insecticides and repellants** (n = 1; 0.1%)	0 (0.0)	0 (0.0)	1 (100.0)
**Respiratory system** (n = 21; 1.8%)	17 (80.9)	0 (0.0)	4 (19.05)
**Sensory organs** (n = 7; 0.6%)	1 (14.3)	2 (28.6)	4 (57.1)
**Various** (n = 2; 0.2%)	1 (50.0)	0 (0.0)	1 (50.0)

Table 6 shows the number and percentage of patients who were prescribed at least one of the drugs licensed, off-label or unlicensed. For all drug groups, the 15 patients who received an unlicensed drug also received one or more licensed drugs as well as at least one off-label drug. Of the remaining patients, 138 received both a licensed and an off-label drug. There were 35 patients whose charts showed that they received an off-label anti-infective as well as a licensed one.

**Table 6:** Number (percentage) of patients who received at least one of the drugs in the given groups.

Drug group	Licensed	Off-label	Unlicensed	None
**Any drug**	178 (93.7)	165 (86.8)	15 (7.9)	0
**Anti-infectives**	58 (30.5)	69 (36.3)	0	98 (51.6)

Of the 190 patients, when the diagnosis was classified into the World Health Organisation (WHO) Major Diagnostic Categories (MDCs), the six most common categories were injury, poisoning and certain other consequences of external causes (n = 33; 17.4%), diseases of the digestive system (n = 31; 16.3%), symptoms, signs and abnormal clinical and laboratory findings, not elsewhere classified (n = 22; 11.6%), neoplasms (n = 14; 7.4%), congenital malformations, deformities and chromosomal abnormalities (n = 14; 7.4%) and diseases of the genitourinary system (n = 13; 6.8%). The number of licensed, off-label and unlicensed drugs prescribed within the six categories is summarised in Table 7. When considering only licensed and off-label drugs prescribed within the six most common MDCs, the proportion of licensed and off-label medications do not appear to differ significantly between diagnostic categories (p = 0.1605)

**Table 7:** Frequency of licensed, off-label and unlicensed drugs prescribed to paediatric patients in the six most common WHO major diagnostic categories.

Major Diagnostic Category		Licensed		Off-label		Unlicensed	
		n	%	n	%	n	%
2	Neoplasms (n = 94)	30	31.9	61	64.9	3	3.2
11	Diseases of the digestive system (n = 284)	121	42.6	156	54.9	7	2.5
14	Diseases of the genitourinary system (n = 89)	45	50.6	44	49.4	0	0
17	Congenital malformations, deformities and chromosomal abnormalities (n = 98)	49	50	49	50	0	0
18	Symptoms, signs and abnormal clinical and laboratory findings, not elsewhere classified (n - = 80)	33	41.3	47	58.8	0	0
19	Injury, poisoning and certain other consequences of external causes (n = 195)	88	45.1	105	53.9	2	1
	All others (n = 316)	149	47.2	161	51	6	1.9

## Discussion

This study has found that more than half of the drugs prescribed were off-label or unlicensed, mainly being off-label (54.0%). This was higher than the 25.7% reported by Czarniak et al. (2015) [1] and another recent Australian study in Tasmania which reported 31.8% [2]. The most common reason for off-label prescribing in this study was indication (59.3%), whereas the aforementioned Australian studies both found that high doses were the most common factor [1, 2]. The current findings show little reduction in the level of off-label prescribing, despite attempts by regulatory agents to reduce it. Such prescribing lacks scientific evidence and regulatory authority. It poses greater risks to patients and a potential for litigation of prescribers [17]. Variations in study design makes a direct comparison between many studies difficult. For example, some studies are retrospective [2, 18, 19], while others are prospective [4, 5, 20–25], in many studies data were collected on all patients admitted to the study ward in a specific time frame over several weeks [22, 24], while in others data were collected on a specific day each month for 12 months [25] or one day each week [20] for a specified time. In another study, medications prescribed to a randomly chosen sample of patients were studied over a 24 hour period [23]. The lengths of studies, the settings and the ages of patients also varied with studies [4, 19, 22].

With respect to the current study, this was compared to an initial study which was carried out five years earlier (in 2008) in the same hospital investigating off-label and unlicensed prescribing in inpatients, outpatients and the Emergency Department. A high level of off-label prescribing was reported across the three settings (25.7%) however, when only inpatients were considered, the level of off-label and unlicensed prescribing was 74.4%. Despite a decrease in off-label and unlicensed prescribing reported in the current study (54.0%), this level is still too high.

The four most frequently prescribed drugs overall were paracetamol, ondansetron, fentanyl and ibuprofen. The most commonly prescribed off-label drugs respectively were ondansetron, fentanyl, oxycodone and paracetamol. Oxycodone, clonidine, parecoxib, and midazolam were always prescribed off-label. Czarniak et al (2015)[1] also reported that ondansetron was the most commonly prescribed off-label drug.

Ondansetron was prescribed off-label on 116 occasions, often for reasons of indication (n = 66; 56.9%) or dose (n = 50; 43.1%). The intravenous formulation (IV) of ondansetron is indicated for the prevention of post-operative nausea and vomiting (PONV) in children aged two to 12 years and the IV or oral formulations are both indicated for emetogenic chemotherapy induced nausea and vomiting in children aged four years and over. Ondansetron was classified as off-label in children receiving chemotherapy because the dose was calculated based on 0.1mg/kg, rather than 5mg/m^2^, as specified in the PI. For PONV, it was often prescribed on a “when required” basis despite the lack of studies evaluating repeat dosing in paediatric patients experiencing PONV [14]. In multiple instances, children were prescribed oral ondansetron for PONV, despite the indications in the PI for emetogenic chemotherapy and radiotherapy only. Similar issues were identified by Ballard et al. (2013)[2].

Paracetamol was often prescribed at doses above the 15mg/kg dose specified in the PI. This was previously reported by Czarniak et al (2015)[1] and Ballard et al. (2013)[2]. Standard reference texts used by many clinicians and pharmacists within Australia, but not the PI, state that paracetamol may be given in doses up to 90mg/kg/day under medical supervision [26, 27], which was the case for all patients in the study. The legal issues surrounding these findings are untested.

Off-label prescribing of antibiotics in paediatrics is common and often warranted, however it is also concerning due to the increase in global antimicrobial resistance [28, 29]. In this study, five antibiotics, cephazolin, amoxicillin, Timentin (ticarcillin and clavulanic acid), flucloxacillin, and gentamicin, were included in the top 20 most frequently prescribed drugs. Cephazolin and Timentin were most frequently off-label due to indication, being only indicated in proven infections, and not for prophylactic or empirical use [14]. Amoxicillin and flucloxacillin were mostly classified as off-label because higher doses than recommended in the PI were prescribed. Similarly to the findings reported by Ballard et al. (2013)[2], gentamicin was often classified as off-label due to single daily dosing, despite the PI stating that divided daily doses should be administered [2, 14]. It has been common medical practice for some time to administer gentamicin once daily due to fewer instances of ototoxicity and nephrotoxicity [30]. Further, in Australia the AMH [26] and the Paediatric Formulary published by the Royal Children’s Hospital in Melbourne [31] recommend once daily dosing. It would appear the PI has not been updated by the sponsor, or any update lacks adequate scientific rigour.

Unlicensed prescribing did not represent a large proportion of prescribing in this study. It consisted of chlorhexidine (the most commonly prescribed unlicensed drug) and several suspension formulations including allopurinol, dexamethasone, hydrochlorothiazide, omeprazole and pantoprazole, which were reformulated from tablets into suspensions. Several commercially unavailable eye drops such as phenylephrine eye drops were also prepared extemporaneously. This arises because of a lack of suitable paediatric formulations.

Although many drugs have been used in an off-label or unlicensed manner for years, there seems to be an unwillingness of drug companies to carry out studies in children to expand their PI’s. Some amendments might be possible based on already published studies subsequent to initial TGA registration. However, for this to occur, the drug company would need to apply to the TGA to update the approved information [2]. Unfortunately, there is little incentive for the drug companies to do this as they will get very little return, notably because children represent a small proportion of the medicine market. Ethical reasons also limit the number of paediatric clinical trials available, as well as lack of parental awareness of their importance [6, 8].

There were several limitations to this study, including that the data were collected some years ago and that new medicines and also new patterns of use of old medicines, could have changed the panorama today. Further, the data were collected retrospectively so there may have been some omissions in data or the data in the medical records may have been recorded incorrectly. In addition, it is possible that there may have been errors in collecting the data although every effort was made to ensure accuracy of data collection. Further, the study was carried out over a one week period and there is a possibility that it may not reflect prescribing in another period or season. The study also did not include neonates. This was because PMH is not a neonatal hospital and only admits children once they have been discharged from a neonatal hospital or if they require intensive care, which was not part of this study. Regardless, from this study and others, it is clear that paediatric prescribing needs to become more evidence based and that the evidence available currently needs to be reflected in published product information and dosage guidelines. Australia has been attempting to improve research in paediatric medicine and improve children’s access to medicines, supporting Australia’s Quality Use of Medicines (QUM) [32]. Although a Paediatric Medicines Advisory Group was formed in Australia and recently, the (AMH) Children’s Dosing Companion [33], which is updated annually, has become available, more should be done to improve the quality of evidence for prescribing off-label and unlicensed drugs in children. The AMH Children’s Dosing Companion [33], which is a useful drug and information reference, is intended to assist practitioners prescribing for children. However, it contains a disclaimer which clearly states that it is not for sale outside Australia, that information may not be consistent with approved PIs, that the authors do not warrant the accuracy of the information contained in the AMH Children’s Dosing Companion and that no responsibility is taken for any loss, damage or injury caused by using the information therein. The efforts of the EMA and FDA are not yet influencing off-label and unlicensed prescription numbers in Australia. These data show even higher levels than others only recently reported. The use of expert groups providing guidance does not affect the PI decisions and partly circumvents the underlying issue. What is also concerning is that despite the high level of off-label prescribing for children, neither the current study or the previous recent Australian studies [1, 2] reported signed consent forms for their use.

Studies by drug companies are needed to evaluate the safety, quality and efficacy of many off-label drugs used in paediatrics. Where unlicensed drugs are reformulated in hospitals, these should be appropriately evaluated by a government sponsored group.
